# Longitudinal Impact of Grade Retention on Student Engagement with School: Challenges to Inclusive Education and to Person-Centered Schools

**DOI:** 10.3390/ejihpe15100213

**Published:** 2025-10-16

**Authors:** Alberta Sousa, Maria João Rodrigues, Mariana Rodrigues, Nadine Poltz, Angeles Conde-Rodriguez, Paulo A. S. Moreira

**Affiliations:** 1 Psicoloxía Evolutiva e Comunicación, University of Vigo, 32004 Ourense, Spain; msousa@alumnos.uvigo.es (A.S.); angelesconde@uvigo.gal (A.C.-R.); 2 Departamento de Educação e Psicologia, Escola de Ciências Humanas e Sociais, University of Trás-os-Montes and Alto Douro (UTAD), 5000-801 Vila Real, Portugal; 3 Center for Research and Intervention in Education, Faculty of Psychology and Education Sciences, University of Porto, 4099-002 Porto, Portugal; mrodrigues@fpce.up.pt; 4 Inclusive Education, University of Potsdam, 14476 Potsdam, Germany; nadine.poltz@uni-potsdam.de; 5 Centro de Investigação em Desporto, Saúde e Desenvolvimento Humano (CIDESD), University of Trás-os-Montes and Alto Douro (UTAD), 5001-801 Vila Real, Portugal; 6 PHI-WELL Research Group, University of Stavanger, 4021 Stavanger, Norway

**Keywords:** student, adolescent, engagement with school, grade retention, longitudinal study, person-centered schools, inclusive education

## Abstract

The value of grade retention as a pedagogic resource remains a subject of debate because of its costs and benefits. In fact, it has been repeatedly argued that grade retention has substantial psychosocial effects. Student engagement with school is one of the dimensions that is expected to be more affected by grade retention. This article aimed to contribute to this debate by examining the relationship between grade retention and various dimensions of student engagement with school. With that purpose in mind, we analyzed data from a sample at one point in time (cross-sectional) and over three points in time (longitudinal), and expanded their meaning for Inclusive Education and for Person-centered schools. The cross-sectional sample comprised 739 students aged 14 to 19 years (M = 16.47, SD = 0.59), while the longitudinal sample included 238 students aged 11 to 15 years (M = 13.29, SD = 0.54 at the first assessment). Student engagement with school was measured using the Multifactorial Measure of Student Engagement. The results indicated that grade retention was negatively associated with overall student engagement. This negative association was particularly evident in the cases of study behaviors and perceived family support for learning. Moreover, students with a history of retention exhibited a significantly steeper decline in engagement over time compared to their non-retained peers. These findings underscore the importance of developing inclusive educational practices. Strategies that foster student engagement are especially relevant for retained students. Schools need to assume their responsibility in promoting positive academic trajectories for all their students, which may require schools shifting from a materialistic-oriented paradigm to a person-centered school paradigm.

## 1. Introduction

Grade Retention—the repetition of a school year—is a practice regularly used in Schools. According to data from the Program for International Student Assessment (PISA), 27% of students in Portugal had repeated a school year at least once during their compulsory education—placing the country above the OECD average in terms of retention rates (organization for Economic Co-operation and Development [OECD], 2018, as cited in European Commission, 2020). Santana (2019) suggested that this trend may be attributed to the national perception of retention as beneficial and its entrenched role within the Portuguese school culture.

### 1.1. Understanding Grade Retention: Academic and Psychosocial Implications

Retention refers to the practice of requiring students who have not met the academic goals for a given school year (Pipa & Peixoto, 2022) to repeat the same instructional content the following year by staying in the same grade for another year (Klapproth et al., 2016; Martorell & Mariano, 2018; Pereira & Reis, 2014). The primary aim is to strengthen students’ understanding of foundational content before progressing to more advanced ones (Martorell & Mariano, 2018). Since the early 20th century, the academic and developmental consequences of retention have been widely studied, particularly in relation to learning outcomes, behavior, and emotional development (Rebelo, 2009). A systematic review and meta-analysis conducted by Goos and colleagues concluded that the effects of retention are mixed, showing both positive and negative developmental impacts for retained and non-retained students (Goos et al., 2021).

There is ongoing debate among researchers regarding the effectiveness of retention as a response to academic underperformance (Borghesan et al., 2022; Martorell & Mariano, 2018; Nunes et al., 2018; Pipa & Peixoto, 2022). Proponents argue that retention may help students overcome learning difficulties (Pereira & Reis, 2014) and achieve expected learning outcomes (Klapproth et al., 2016), as it provides additional time to consolidate foundational knowledge before advancing (Borghesan et al., 2022). Additionally, retention has been associated with higher homogeneity in terms of academic performance in the classroom (Klapproth et al., 2016).

In contrast, researchers have noted the financial costs of supporting an additional year of schooling and the delayed entry of students into the labor market (Borghesan et al., 2022; Pereira & Reis, 2014). Despite these more economical considerations, research has linked retention to adverse psychosocial outcomes for retained students, including reduced self-esteem, impaired peer relationships (Borghesan et al., 2022; Pereira & Reis, 2014), perceived distance from school, a higher likelihood of dropping out of school (Pereira & Reis, 2014), disruptive behavior in classroom (Pagani et al., 2001), and increased risk of stigmatization by peers (Borghesan et al., 2022).

The impact of retention on students’ academic performance appears to vary over time. Initially, retained students may exhibit improved academic outcomes (Klapproth et al., 2016; Nunes et al., 2018; Pereira & Reis, 2014). However, these preliminary positive effects are not sustained in the long term, becoming negligible (Klapproth et al., 2016; Nunes et al., 2018) or even adverse (García-Pérez et al., 2014; Hwang & Cappella, 2018; Pereira & Reis, 2014). Notably, Borghesan et al. (2022) identified some positive long-term effects in math and Portuguese for most of the studied students, although almost one-third did experience a learning loss in the long term. Given that retention is typically a response to prior difficulties in meeting academic goals (Martorell & Mariano, 2018), this practice needs to be overthought in light of these adverse results. It seems essential to evaluate the effects of grade retention not only in terms of academic performance but also in light of broader psychosocial factors such as students’ emotional well-being and self-perception (Nunes et al., 2018). Recent research has increasingly examined these psychosocial dimensions in the context of grade retention.

N. Santos et al. (2022) highlighted that retained students reported diminished perceptions of their value as students. Conversely, these students did not indicate lower levels of well-being or school belonging (N. Santos et al., 2022), which contrasts with findings from previous studies (Pipa & Peixoto, 2022; Van Canegem et al., 2021). In contrast, Hwang and Cappella (2018) and Klapproth et al. (2016) found no significant correlation between psychosocial variables and retention, except for a negative association with self-concept levels among seventh-grade students (Klapproth et al., 2016). Pipa and Peixoto (2022) concluded that retained students exhibited reduced task orientation, sense of belonging, and perceived value of school. Moreover, retained students demonstrated less interest in pursuing higher education (N. Santos et al., 2022) and held lower expectations regarding their academic development (Flores et al., 2013).

### 1.2. The Crucial Role of Student Engagement with School for Educational Outcomes

While academic performance and psychosocial factors are critical in evaluating the impact of grade retention, they do not fully capture how students relate to school on a daily basis. In addition, indicators such as suspension and absenteeism rates offer only a partial view. For example, Martorell and Mariano (2018) found no statistically significant long-term effects of grade retention on these variables, while Gubbels et al. (2019) reported a small increase in absenteeism and a substantial increase in dropout rates associated with previous retention. Although important, such behavioral metrics are insufficient for fully understanding students’ connection to school. In light of this, Martorell and Mariano (2018) recommend incorporating socio-emotional dimensions and the quality of students’ relationships with teachers and peers when assessing the consequences of grade retention. Within this broader perspective, student engagement with school emerges as a key indicator—distinct from grades or self-perception.

Although there is no consensus about conceptualization of student engagement with school, growing evidence have conceptualized student engagement with school as a multidimensional construct, with some authors testing integrative frameworks of the constructs. For example, Moreira and colleagues tested the integration of the items and dimensions of two of the most disseminated assessment instruments’ (Moreira et al., 2020a) and found that a multifactorial structure for the construct of student engagement registered good validity in several indicators. On the one hand, they found support for the integration of individual and contextual dimensions in the same factorial structure; on the other hand, this factorial structure was sensitive to capturing the associations between each of the dimensions and both student academic performance and subjective well-being (Moreira et al., 2020a). Individual dimensions include emotional, cognitive, conduct, and study behaviors, and the contextual dimensions include teachers, family, and peers’ support for learning, being consistent, and integrating the more consensual frameworks (e.g., Reschly & Christenson, 2022). The four individual characteristics of student engagement with school are distinctly characterized and refer to different aspects of the students’ experience of school. Emotional engagement encompasses affective reactions to school, including a sense of belonging and identification with the institution; cognitive engagement refers to representations and beliefs about school; and study behaviors refer to study strategies and involvement in school work (Fredricks et al., 2004; Moreira et al., 2020a; Reschly & Christenson, 2022). The dimensions of family, teachers, and peers’ support for learning refer to students’ perceptions about the support for learning they receive from each of these interpersonal structures (Appleton et al., 2006; Fredricks et al., 2004). Student engagement with school emerges through the dynamic interactions between the individual and the contextual characteristics.

Engaged students tend to demonstrate a sense of connection to the educational environment, experience positive emotions in the classroom, and perceive their schoolwork as relevant to achieving future goals. Consequently, they tend to employ adaptive cognitive strategies to facilitate their learning (Kretschmann et al., 2019). Furthermore, student engagement with school is a strong predictor of academic processes and outcomes (e.g., Caldeira et al., 2013) and serves as a facilitator of academic adaptation (Hirschfield & Gasper, 2011; C. Silva et al., 2021; S. L. R. Silva et al., 2016). Notably, the cognitive dimension of engagement, as opposed to the emotional dimension, seems to significantly influence academic performance (Szabó et al., 2024). However, other researchers have indicated that higher levels of behavioral engagement can predict improved academic grades (Chase et al., 2014) and lower dropout rates (Wang & Fredricks, 2014).

In contrast, low or inconsistent levels of student engagement with school may correlate with disruptive behaviors, low academic performance, and the teacher’s perception of diminished behavioral engagement (Archambault & Dupéré, 2016). Additionally, such students often experience conflicting relationships with their teachers (Archambault & Dupéré, 2016). Furthermore, declining levels of behavioral and emotional engagement have been linked to misconduct, substance use, and delinquency in subsequent years (Demanet & Van Houtte, 2013; Wang & Fredricks, 2014).

An important quest facing science is how scientists’ practice and science production contributes to building trust in science, as this is a paramount aspect for the relations between science and society (Petousi & Sifaki, 2020). It is fundamental, then, that scientific findings are put in the context of societies’ realities so they can inform how they can contribute to the societies’ positive development. Consistently, there is a growing consensus about the need for educational policies and school practice to rethink student grade retention (Bowman, 2005; Ferrão, 2015; Hwang & Cappella, 2018; Larsen & Valant, 2023; Simpson, 2005; Tingle et al., 2012; Valbuena et al., 2021). On the one hand, the iatrogenic effects of grade retention are frequently more substantial than its benefits. On the other hand, contemporary approaches to education and human development claim more and more for individualized and person-centered approaches that acknowledge and consider the complex dynamics among the different dimensions involved in student–context interactions. This need is well described in the following:

*“Technological and material resources are available for humans at an unprecedented level, and yet a significant percentage of the population report some degree of subjective suffering, functioning impairment, or medical ill-being associated with patterns of maladaptive psychosocial functioning/lifestyles.*

*This suggests that there is a vital need for new approaches to promoting human development. School is one of the most powerful contexts for implementing such approaches. However, a new paradigm in education is required to help schools be more efficient at preparing their students to deal adaptively with the challenges facing humanity. Schools need to be able to promote the processes underlying human holistic development, rather than emphasizing the development of mainly logical-propositional dimensions, as is the case of materialistic-oriented conventional schools (…)*

*School is an ideal context for implementing a holistic approach to the promotion of human functioning. However, the effectiveness of any means aiming to promote positive adaptation in (person-centered) schools depends on intentionality, coordination, systematization, continuity, evaluation, and monitoring. We need to develop and test coherent frameworks that describe the common factors, and dynamics amongst them, involved in changing conventional schools to person-centered schools. This process is in its embryonic phase and is one of the current main challenges for research and practices of behavioral sciences. If done effectively, it will have substantial implications, not only for individuals’ well-being, but also for societal organization and development”.*

(Moreira & Garcia, 2019, pp. 183–184)

### 1.3. A Complex Interplay Between Student Engagement and Grade Retention

Researchers such as Mahatmya et al. (2012) have suggested that retention may negatively impact student engagement with school. This assumption is supported by research conducted in Portugal (Gandra & Cruz, 2021). However, it is crucial to recognize specific limitations inherent in research on student engagement with school, particularly regarding the diverse constructs and dimensions employed across previous studies. For instance, Demanet and Van Houtte (2013) identified increased rates of misconduct among students with a history of retention. However, their study did not account for other dimensions of student engagement, thereby providing an incomplete picture of the broader engagement context. These discrepancies can hinder the comparison of results across different studies and complicate an integrated understanding of the relationship between retention and student engagement with school across its various dimensions.

Additionally, most studies that explore the relationship between grade retention and student engagement with school have captured this interaction at a single point in time, thereby limiting the ability to understand causal relationships. Consequently, there is a pressing need for longitudinal studies that track students over time to better ascertain the effects of retention on both academic and psychosocial development (N. Santos et al., 2022). This need is particularly pertinent in Portugal, where few studies have been conducted on this topic despite high retention rates compared to other European Union countries (Pipa & Peixoto, 2022).

The main aim of this article was to deepen our understanding of the associations between grade retention and student engagement in light of a conceptual framework that encompasses the various dimensions of student engagement with school described in the literature. Thus, we revisited the study’s results reported by M. A. S. M. Santos (2023), where the primary research question was as follows: What is the relationship between grade retention and students’ engagement with school? In order to answer that question, the following cross-sectional and longitudinal examinations were performed:

1. Cross-sectional examination: This part of the study examined the association between grade retention and the multiple dimensions of student engagement with school at a single time point. Based on Mahatmya et al. (2012) and Gandra and Cruz (2021), it was hypothesized that retention would be negatively associated with student engagement with school across its various dimensions.

2. Longitudinal examination: In addition, the study investigated the impact of grade retention on student engagement with school over time. It was hypothesized that grade retention would negatively affect engagement longitudinally, accentuating a trend of diminishing engagement with school as students advanced academically.

Cross-sectional and longitudinal methodologies provide a robust framework to comprehensively assess the short-term and long-term impacts of retention on students’ engagement with school. Moreover, they have the potential to favor the understanding of the grade retention–engagement phenomenon from an inclusive education and person-centered point of view.

## 2. Materials and Methods

The first two measurement points of the longitudinal data in the study have been previously detailed in earlier works on student engagement with school. Nevertheless, grade retention has never been linked within this data to student engagement with school so far.

### 2.1. Participants

Data was collected in a sub-sample of schools that were participating in a larger longitudinal study in Portugal. Schools participating in that larger longitudinal school were selected based on the national territorial distribution of schools, which was the criterion of strata for school sampling. From these, schools whose students participated in the data collection in all the data collection moments described in this article, were included. 53.58% (*n* = 396) of the students were from rural schools, and 46.42% (*n* = 333) were from urban schools. Students in each sample were those whose parents gave consent for their children to be included in each moment.

The cross-sectional examination included a total sample of 739 students from the 9th grade (*n* = 23), 10th grade (*n* = 89), and 11th grade (*n* = 615), with 42.8% male and 57.2% female participants, aged between 14 and 19 years (M = 16.47; SD = 0.59). Regarding maternal education, 56.0% held a degree lower than secondary education (<twelfth grade), 25.2% finished secondary education, and 18.8% held a degree higher than secondary education. From the total sample of 739 participants of the transversal study, 18.6% were retained students. Among these retained students, 59.1% were male, with ages ranging from 14 to 19 years old (M = 16.8; DP = 0.79). Among retained students, at the moment of the data collection 23 students were in the 9th grade, 77 in the 10th grade and 28 in the 11th grade. Regarding the school level when students were retained, 23 were retained in “high school”, 89 in “middle school” and 15 in “elementary school”.

Within the longitudinal data set, the sample analyzed included only those students who completed the surveys at all four measurement points. In total, 238 students (61.3% female and 38.7% male) aged between 11 and 15 years (M = 13.29; SD = 0.54) at first assessment (M0) and between 16 and 20 years (M = 17.19; SD = 0.49) at the last assessment (M3) were incorporated. During the first data collection, all students were in the seventh grade, and by the final data collection, three were enrolled in the ninth grade, 34 in the eleventh grade, and 201 in the twelfth grade. Regarding maternal education, 56.4% held a degree lower than secondary education (<twelfth grade), 25.5% finished secondary education, and 18.1% held a degree higher than secondary education.

### 2.2. Instruments

To assess student engagement with school, the Multifactorial Measure of Student Engagement (MMSE, Moreira et al., 2020a) was used. This measure assesses seven dimensions of student engagement with school through 27 items, incorporating both individual and contextual dimensions. Student responses were recorded using a four-point Likert-type scale, ranging from 1 (Strongly Disagree) to 4 (Strongly Agree. This measure demonstrates strong psychometric properties, particularly in terms of structural validity (CFI = 0.954, RMSEA = 0.035, SRMR = 0.037) and internal consistency, both for the overall scale (ω = 0.93) and for specific dimensions including student conduct (ω = 0.82), study behaviors (ω = 0.80), cognitive engagement (ω = 0.73), emotional engagement (ω = 0.77), teacher support for learning (ω = 0.73), family support for learning (ω = 0.73), and peer support for learning (ω = 0.78. Furthermore, it also shows indicators of convergent validity between student engagement and academic performance (r = 0.21, *p* < 0.001) as well as emotional well-being (r = 0.56, *p* < 0.001) (Moreira et al., 2020a).

Grade Retention was assessed using the academic records of students available at the schools.

Sociodemographic characteristics regarding information on the student (age, gender, school year) and the students’ parents (mother’s education) were assessed during the survey. Mother’s education was scored from “1” = fourth-grade educational level to “9” = post-doctorate educational level.

### 2.3. Procedures

Schools were contacted by the researchers of the study, the study’s objectives and procedures were presented, and schools were invited to participate. In those schools that accepted to participate, the objectives and the procedures of the study were presented to students’ guardians. Students, guardians, and students were invited to participate. An informed consent form was delivered to students’ guardians who accepted to participate in the study. For those students whose guardians delivered the signed informed consent back to the schools, schools and researchers scheduled the moments for data collection. Data was collected by a researcher who went to the schools. Students whose guardians gave informed consent for their youth to participate were grouped in classrooms in their schools. The surveys were given to students in paper format and students filled in the surveys in groups, with the supervision of a researcher who monitored the data collection procedures in person. Students in each sample were those whose parents gave consent for their children to be included in each moment. All schools, students and families contacted in the first moment were also contacted in the other moments. Within the longitudinal data set, the same group of seventh graders was observed at four distinct time points over a period of five years. There was a one-year gap between the first (M0) and the second measurement point (M1), a two-year gap between the second and third measurement point (M1 and M2), and one year between the third and fourth measurement point (M2 and M3). We included in the longitudinal sample and in the longitudinal analyses only the students who, besides having been included in the transversal study, had also participated simultaneously in the several moments of data collection (*n* = 241).

### 2.4. Data Analysis

#### 2.4.1. Cross-Sectional Examination

To investigate the effect of grade retention on components of student engagement with school, a structural equation modeling (SEM) analysis was conducted using IBM SPSS Amos 26.0 (International Business Machine Corporation, 2019). This multivariate analysis technique enabled the exploration of pathways and measurement models, as well as the examination of external predictors that could explain variability (Marôco, 2010). In this study, gender (0 = male; 1 = female), age, grade level, and maternal education were incorporated into the model as control variables.

#### 2.4.2. Longitudinal Examination

To explore the effect of grade retention on the overall scale of student engagement with school and its seven dimensions, both at the initial time point and over time, a series of latent growth model analyses were conducted using IBM SPSS Amos 26.0 software (International Business Machine Corporation, 2019). These models are a specific application of structural equation modeling that allows for the consideration of both intraindividual changes in behavior over time and interindividual differences in these changes (Marôco, 2010). According to Marôco (2010), this statistical technique also facilitates the analysis of external predictors that may explain variability, both in terms of initial values (intercept) and change trajectories (slope). To address missing data, the maximum likelihood method was adopted, as it is considered a suitable approach for latent growth modeling (Marôco, 2010).

The latent growth models were initially conducted using all four observation points. However, due to results that did not yield admissible solutions or minimally acceptable model fit quality, the first measurement point (M0) was excluded from the analyses, leaving only three observation points for longitudinal examination with M1 referred to as the initial time point in all subsequent analyses.

The data analysis was conducted in two phases. To examine variation in both grouped terms (fixed effects) and individual terms (random effects) for the overall scale of student engagement with school and its seven dimensions across the three observation points, three unconditional latent growth models were initially used. For model identification, it was assumed that the slope was zero at the initial time point (M1) and that there was a linear growth tendency thereafter. The path weights between the slope and the manifest variables were set at 0, 0.66, and 1, respectively. The latent intercept variable was included in the models to examine the average value of the dependent variables at the three observation points, with all paths from the intercept to the dependent variables fixed at a weight of 1. The mean of the intercept and slope enabled the determination of the average starting values of the dependent variables and their average rate of change over time. The variances of the intercept and slope were used to assess individual differences in both baseline values and the rate of change in student engagement with school and its seven dimensions. In the second phase, conditioned models were estimated, in which retention variables at M2 and M3, as well as control variables (gender, age, school year, and mother’s education) assessed at all three measurement points, were included as predictors for the intercept and slope. Effects from independent variables at later observation points were not regressed onto earlier ones, as they could not have exerted any influence.

To address missing data, the maximum likelihood method was employed, deemed suitable for latent growth modeling (Marôco, 2010). To assess the fit quality of the model, the Chi-square test (Chi^2^/df), Comparative Fit Index (CFI), Tucker–Lewis Index (TLI), and Root Mean Square Error of Approximation (RMSEA) were utilized (Marôco, 2010). Thresholds for good model fit were established as Chi2/df < 5, TLI and CFI > 0.90, and RMSEA < 0.08 (Arbuckle, 2008).

## 3. Results

### 3.1. Cross-Sectional Results

**Figure 1:**
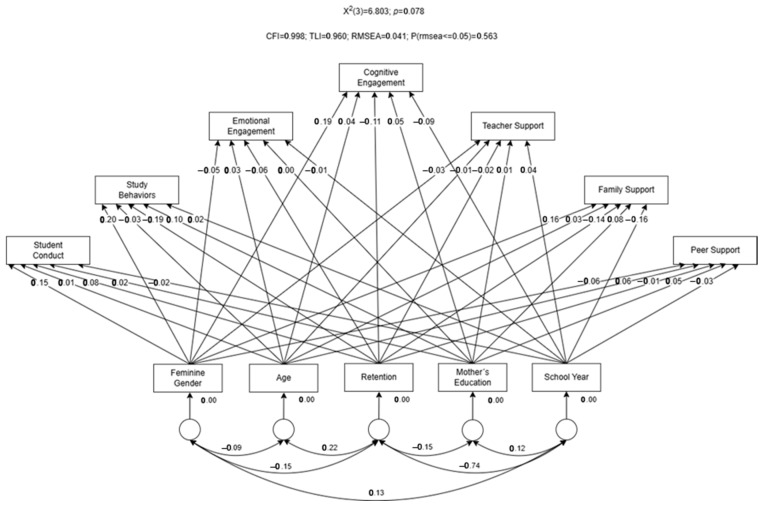
Structural equation model describing the effect of gender, age, retention, mother’s education, and school year on each one of the seven dimensions of Student Engagement with school. Adapted from: M. A. S. M. Santos (2023). Retenção Escolar e Envolvimento dos Estudantes com a Escola: desafios para a educação inclusiva. Doctoral Dissertation. University of Vigo, Ourense Campus. Unpublished Dissertation.

Figure 1 illustrates the analyzed model. Based on modification indices (MI) > 4 (*p* < 0.001), nonsignificant correlations between the error terms of dependent variables were removed, and some measurement errors in the dependent variables were correlated to enhance the model’s fit to the data, as recommended by Marôco (2010).

Table 1 summarizes the standardized coefficients and corresponding *p*-values for the model testing the effect of retention on the seven dimensions of student engagement with school, while controlling for sociodemographic variables (gender, age, school year, and mother’s education). The results indicated that retention is negatively correlated with study behaviors (r = −0.188; *p* < 0.001) and family support (r = −0.139; *p* = 0.015). Regarding the control variables, it was found that female students tended to report a more positive perception of student conduct (r = 0.155; *p* < 0.001) and study behaviors (r = 0.197; *p* < 0.001), as well as higher levels of cognitive engagement (r = 0.189; *p* < 0.001) and family support (r = 0.164; *p* < 0.001), compared to male students.

### 3.2. Longitudinal Results

Results of descriptive analysis for student engagement variables across the three measurement points used for analysis are summarized in Table A1, Appendix A. Preliminary analyses indicated that the study variables exhibited absolute values of skewness and kurtosis below 3 and 10, respectively, which supports the assumption of multivariate normality (Kline, 2005). Based on modification indices (MI) > 4 (*p* < 0.001), some measurement errors in the dependent variables were correlated to achieve a better fit of the model to the data, following the recommendations of Marôco (2010).

#### 3.2.1. Results of Unconditional Latent Growth Models

The unconditional latent growth models demonstrated good model fit, with the exception of the models for student conduct and emotional engagement.

Table 2 summarizes the results of the unconditional models for the overall scale of student engagement with school and its seven dimensions, presenting the unstandardized estimates (and corresponding standard errors) for both the mean (fixed effect) and variance (random effect) of the intercept and slope.

As noted, the intercept mean reflects the initial average levels of the dependent variables, while its variance indicates whether individual differences exist in both the initial values and the rate of change in student engagement with school across its seven dimensions.

The results show that both the mean and the variance of the intercept were statistically significant. This suggests that there was interindividual heterogeneity in students’ initial levels of the dependent variables.

The mean and variance of the slope provided insight into the average rate of change in the dependent variables over time (fixed effects), as well as the existence of individual differences in these change trajectories (random effects). The results showed significantly negative mean slopes for the overall scale of student engagement with school and the dimensions of cognitive engagement, emotional engagement, student conduct, and peer support, suggesting a slight decline in their average values throughout the study period. In contrast, the mean slopes for study behaviors, teacher support, and family support were not statistically significant, indicating no significant changes in these components over time.

With regard to the random effects, the variance of the slopes for the two dimensions, student conduct and teacher support, was not statistically significant, indicating that participants did not differ in their individual change trajectories. For the remaining dependent variables, however, slope variances were statistically significant, revealing interindividual variability. Given this scenario, it was deemed relevant to include additional predictor variables in the models, specifically the predictor variable retention, along with control variables (age, gender, mother’s education level, and school grade level), to help explain the observed heterogeneity in participants’ baseline values and change patterns over time.

With the exception of student conduct and teacher support, all other dependent variables showed statistically significant and negative correlations between intercept and slope. This indicates that students with higher initial levels of student engagement with school (as a composite variable), or in the dimensions of cognitive engagement, emotional engagement, study behaviors, family support, and peer support, tended to exhibit steeper declines over time. Conversely, students with lower initial levels tended to show slower declines or less pronounced negative slopes over time.

#### 3.2.2. Conditional Latent Growth Models

Following the estimation of the unconditional latent growth models, control variables (female gender, age, school year, and mother’s education) and the predictor variable (retention) at all three measurement points were introduced as predictors of the initial levels (intercepts) and growth trajectories (slopes) of the dependent variables.

When running the models with all control and predictor variables observed across the three measurement points, the results revealed a high correlation between variables at different moments—for example, between retention at M2 and M3. This constrained the analyses and led to the decision to only include one measurement point per variable. One severe outlier was removed, reducing the final sample to 238 participants. The proposed models demonstrated good fit, with the exception of those for student conduct, teacher support, and family support.

As shown in Table 3, retention emerged as the only significant predictor of the slope of the overall scale of student engagement with school, indicating that retained students exhibited a statistically significant decrease in engagement over time compared to their non-retained peers. When examining the specific dimensions of student engagement with school, retention was the strongest predictor of change in study behaviors over time, with retained students showing a significant decline in this dimension of student engagement with school compared to non-retained students.

Gender was a significant predictor of the initial levels of student conduct—female students began with significantly higher levels than male students.

Regarding study behaviors, the control variables gender, age, and school year predicted the slope of this dimension: female students consistently demonstrated higher levels of study behavior over time than male students. Additionally, students with higher age and grade level at M2 exhibited significantly steeper declines in study behavior over time than younger students or those in lower grades.

Regarding cognitive engagement, gender was a significant predictor, with female students reporting higher levels of cognitive engagement over time compared to male students.

School year (observed at M2) predicted the slope of peer support: as students progressed to higher grades, their perception of support from peers increased. However, gender was also a significant predictor, with female students reporting lower levels of perceived peer support over time compared to their male peers.

For the dimensions of emotional engagement and teacher support, none of the independent variables included were statistically significant predictors of either the intercepts or slopes.

## 4. Discussion

The aim of this article was to examine the relationship between grade retention and students’ engagement with school. To achieve this aim, we examined the results described by M. A. S. M. Santos (2023), including 1. cross-sectional results and 2. longitudinal results, which remained unpublished until now. First, the cross-sectional study was used to examine the association between retention and students’ engagement with school in a large sample of secondary students in Portugal. Subsequently, the longitudinal data set was used to analyze the effect of retention on the overall scale of student engagement with school and its seven dimensions over time. Gender, age, mother’s educational level, and school year were included in the model as control variables.

The hypotheses initially proposed in this study, supported by previous research, appear to have been confirmed by the results. Gandra and Cruz (2021) identified grade retention as a direct contributor to decreased school engagement in Portugal. Similarly, Pagani et al. (2001) found in a longitudinal sample of 1830 elementary school students in a Canadian province, negative effects of retention on academic performance and behavior, both in the medium and long term. Retention was perceived as a marker of failure, frustration, humiliation, shame, and other negative emotions (Pagani et al., 2001).

### 4.1. The Role of Grade Retention for Students’ Engagement with School

Students who had repeated a grade reported lower levels of study behaviors and family support. The literature suggests that family support has a significant effect on school engagement. Specifically, Santana (2019) noted that students encouraged by their parents to make choices about school relationships were more engaged than those who did not receive such parental encouragement. Similarly, Lahaye et al. (2001), as cited in Santana (2019), pointed out that how young people perceived parental involvement in their school life influenced their views on both school and family relationships.

The results of the longitudinal examination were consistent with previous research, indicating that retained students experienced a statistically significant decline in student engagement with school over time compared to non-retained students. Specifically, Gandra and Cruz (2021) conducted a study examining student engagement with school and retention in Portugal, concluding that retention in primary and middle school is associated with lower engagement. Similarly, Mahatmya et al. (2012) explored the relationship between retention and engagement, finding a negative correlation between these variables.

Retention emerged as a key predictor of changes in study behaviors over time. Retained students exhibited a significant decrease in this dimension of student engagement with school compared to non-retained peers. This finding aligns with those of N. Santos et al. (2022), who reported lower values in the behavioral engagement dimension among retained students. Furthermore, Wang and Fredricks (2014) found that students with higher academic success, as measured by grades, demonstrated elevated levels of behavioral engagement.

Given the results of previous studies, which show that retention is associated with a small increase in absenteeism and a substantial increase in school dropout rates (Gubbels et al., 2019), the decreased engagement with school of retained students might be one plausible explanation that needs further investigation. The negative impact of retention on study behaviors over time found by these results is consistent with recent evidence on student engagement and study behaviors as narrow expressions of biopsychosocial organizations. On the one hand, student approaches to learning (which include study behaviors) emerge are behavioral expressions resulting from the integration of the major systems of learning and memory: associative learning and procedural memory, propositional leaning and semantic memory and learning by insight and auto-biographic memory, the very same systems underlying the organization and differentiation of personality (Moreira et al., 2021a). Items capturing study behaviors of student engagement measure used in the study (the Multifactor Measure of Student Engagement—MMSE (Moreira et al., 2020a), which is fully described in Appendix B) are formulated in a way that high values correspond to student’s deep approach to learning and low values correspond to surface approach to learning.

Students presenting a deep approach to learning approach the learning task with the intention of maximizing intellectual understanding and extracting meaning from the task, guided by intrinsic motivation to learn, and they employ strategies characterized by the establishment of relations between specific content and broader phenomena. Students presenting a surface approach to the learning task, characterized by low investment and low effort, are guided by extrinsic motivation to the task. Adopting a surface approach to learning typically has a detrimental impact on academic performance (Diseth, 2013; Moreira et al., 2021a).

Items capturing study behaviors of student engagement measure used in the study (the Multifactor Measure of Student Engagement—MMSE (Moreira et al., 2020a), which is fully described in Appendix B) are formulated in a way that high values correspond to student’s deep approach to learning and low values correspond to surface approach to learning.

A deep approach to learning is more prevalent in individuals having a steady temperament profile (low novelty seeking and high persistence), and having high levels of both self-directedness and cooperativeness (regardless of their self-transcendence, i.e., the SCT “creative” and SCt “organized”profiles) (Moreira et al., 2021b). This finding is noteworthy because temperament-character organizations are modulated by learning, therefore, they are changeable and amenable to intervention, including school experiences. Consistently, the personality dimensions, temperament, and character profiles underlying student approaches to learning are consistent with those underlying the different dimensions of student engagement with school. In a study describing the personality-student engagement interactions, the authors found that temperament steady profiles and character organized and creative profiles were characteristic of engaged students (Moreira et al., 2020b).

Altogether, this evidence helps to understand the findings that grade retention has a negative impact on student study behaviors over time. On the one hand, having unsteady or dysregulated temperament profiles and apathetic character profiles (that are characteristic of students presenting low levels of engagement with school and of students adopting surface approaches to learning place students at risk for grade retention, which may help to understand the episodes of retention of these students. On the other hand, the experience of being retained may be processed differently by different students, but it is very likely that students process the experience of grade retention as an indicator of his/her self-value, about the future, and about the school. Students presenting dysregulated temperament and character profiles and apathetic character profiles are particularly sensitive to experiences that may be a threat to self-worth and, as a consequence, to feel those experiences with negative affect and to decrease the interest and investment in those experiences, such as the case of study behaviors. This highlights the need for schools to adopt a person-centered approach, especially for retained students, so school interventions that are efficient in reversing the tendency for maladaptive study behaviors may be designed and implemented (Moreira & Garcia, 2019).

When analyzing control variables, female students tended to report more positive perceptions of their student conduct and study behaviors, as well as higher levels of cognitive engagement and family support, compared to male students. Additionally, gender emerged as a significant predictor in cognitive engagement, with female students reporting higher levels of cognitive engagement over time compared to male students. It is important to note that previous studies with Portuguese samples (Nunes et al., 2018; Pereira & Reis, 2014) showed higher retention rates among boys, highlighting the importance of examining the relationship between retention and other variables under the control of students’ gender. Furthermore, gender showed statistical significance as a predictor of the slope of peer support, with female students reporting lower levels over time compared to male students.

A note of caution is required in the interpretation of these results. Although studies try to control bias, several studies have shown that selection bias is still a critical factor in retention research (Provencher & Kassel, 2019).

### 4.2. Implications

Studies examining the intersection of inclusion with retention and student engagement with school are almost nonexistent. In particular, students’ opinions and experiences with retention are scarce, with most of the available perspectives in the literature coming from teachers or individuals associated with them. The current article gives an important insight into retention and students’ engagement with school. However, the Portuguese legislative framework concerning school inclusion (Decree-Law No. 54/2018 of July 6) is still new and still developing. Therefore, it is recommended that future studies, particularly in Portugal, continue to explore the effects of inclusive practices that promote academic success, as suggested by authors like Nunes et al. (2018).

The main aim of repeating a grade is to strengthen the understanding of academic content for those students failing to reach the academic goals (Martorell & Mariano, 2018). Consistent with other researchers studying the effects of retention among Portuguese students (Borghesan et al., 2022; Nunes et al., 2018; Pereira & Reis, 2014), these results emphasize the importance of modifying the application of retention practices in cases of academic failure. Nunes et al. (2018) and Borghesan et al. (2022), for instance, recommended adjusting the criteria for applying retention to target students with the lowest grades, thereby reducing the overall number of students retained. But reducing the overall number of students repeating a grade will not help retained students overcome the psychosocial problems associated with retention shown in the literature (e.g., Borghesan et al., 2022; Goos et al., 2021). Pereira and Reis (2014) argued for the necessity of complementing the practice of retention with additional strategies and educational interventions to enhance student performance, especially for those retained in the early years of education. The results examined in this article suggest that these strategies might not only concern academic performance but also different dimensions of student engagement with school. This could help reduce absenteeism and dropout rates in the long term.

## 5. Conclusions

### Challenges for Inclusive Education and for Person-Centered Schools

Results examined in this article reveal that retained students showed significantly lower levels of student engagement with school compared to their non-retained peers. By showing the negative effects of retention on student engagement, this article underscores the critical need for reevaluating retention practices at Portuguese schools. This leads to the claim of shaping educational policies and designing interventions that actively promote student engagement with school and reduce the reliance on retention as a remedial strategy. Furthermore, these findings highlight the potential long-term consequences of retention, emphasizing the importance of proactive measures to prevent the need for retention and avoid school drop-out or placement in Special Education. Such insights contribute to the ongoing dialog about effective strategies for fostering inclusive and supportive school environments that cater to the diverse needs of all students (Decree-Law No. 54/2018 of 6 July).

Considering the research that we have been conducting on student engagement with school (and its role in student academic processes and outcomes and adolescents’ positive development), the results described in this article have implications for educational and societal in several ways.

First, student engagement with school is a process that emerges from the interaction between individual dimensions and contextual influences. Individual dimensions relevant for the process of development of student engagement with school—such as emotions, cognitions, and personality (Moreira et al., 2021a)—become more differentiated and organized as individuals experience the world and organize their experience through the different systems of learning and memory.

Second, student engagement is also a result, as it emerges from the interaction of the individual characteristics and the contexts, including families and schools. In other words, the level of engagement with the school of a student in a specific time at a specific school is also an indicator of the quality of the cumulative experiences of that student with the school. In fact, the quality of such experiences is dependent on the interactions between the individual and contextual dimensions involved in students’ experiences with school. Engagement as an outcome reflects how well or poorly schools succeed in offering their students the necessary conditions for having school experiences that meet psychological needs and keep all their students involved with school.

Student engagement with school is a multidimensional phenomenon, with evidence suggesting measurement invariance in different societies and school levels (Moreira & Dias, 2019). Student engagement with school is a narrow expression of psychobiological organizations (Moreira et al., 2021a), which is consistent with existing frameworks of the typical stage sequence underlying behavioral change. In a study applying the transtheoretical model of change to academic trajectories, Moreira and colleagues found marked and significant differences in personality dimensions and in student engagement with school among the pre-contemplation, contemplation, preparation, and action stages of change in academic performance (Moreira et al., 2020b). However, evidence about individual dimensions does not preclude the influence of contextual factors on the retention–engagement interactions. On the contrary, the understanding of the retention–student engagement with school interaction is only possible when considering the individual-context interaction. As a biopsychosocial organization, student engagement with school is shaped by interpersonal and contextual influences. Family socio-economic characteristics are structural factors in student academic trajectories, with students coming from families with lower socio-economic status having a higher risk for grate retention, but also being at higher risk of experiencing a more negative impact of grade retention on student engagement with school over time (e.g., González-Betancor & López-Puig, 2016; Jimerson & Ferguson, 2007; Nieto-Isidro & Martínez-Abad, 2023). Family resources influence the support and the investment they make in their children’s academic trajectories, which increases or buffers the long-term impact of grade retention engagement with school (Lee-St. John et al., 2018).

In summary, schools need to assume their responsibility in promoting positive academic trajectories for all their students, including shifting from a materialistic-oriented paradigm to a person-centered school paradigm. Person-centered schools are contexts where all the dimensions of holistic functioning are considered and systematic promotion of the dimensions identified by robust evidence as crucial for student’s holistic positive development. Assuming their responsibility from a person-centered school perspective implies that “*educators consider each student’s complete personal story and to envision each individual’s future when deciding upon retention*.” (Smith & Herzog, 2014).

## Figures and Tables

**Table 1:** Standardized regression coefficients for the effect of retention on each one of the seven dimensions of student engagement, while controlling for socio-demographic variables.

			Coefficient	*p*
Student Conduct	<---	Gender_Female_	0.155	<0.001
Study Behaviors	<---	Gender_Female_	0.197	<0.001
Emotional Engagement	<---	Gender_Female_	−0.051	0.169
Cognitive Engagement	<---	Gender_Female_	0.189	<0.001
Teacher Support	<---	Gender_Female_	0.028	0.453
Family Support	<---	Gender_Female_	0.164	<0.001
Peer Support	<---	Gender_Female_	−0.059	0.115
Student Conduct	<---	Age	0.007	0.846
Study Behaviors	<---	Age	−0.030	0.413
Emotional Engagement	<---	Age	−0.026	0.504
Cognitive Engagement	<---	Age	0.039	0.304
Teacher Support	<---	Age	−0.014	0.728
Family Support	<---	Age	0.027	0.473
Peer Support	<---	Age	−0.017	0.671
Student Conduct	<---	Retention	−0.079	0.169
Study Behaviors	<---	Retention	−0.188	<0.001
Emotional Engagement	<---	Retention	−0.061	0.298
Cognitive Engagement	<---	Retention	−0.105	0.065
Teacher Support	<---	Retention	0.058	0.324
Family Support	<---	Retention	−0.139	0.015
Peer Support	<---	Retention	0.012	0.841
Student Conduct	<---	Mother’s Education	0.018	0.616
Study Behaviors	<---	Mother’s Education	0.095	0.007
Emotional Engagement	<---	Mother’s Education	−0.003	0.938
Cognitive Engagement	<---	Mother’s Education	0.045	0.214
Teacher Support	<---	Mother’s Education	−0.013	0.728
Family Support	<---	Mother’s Education	0.078	0.033
Peer Support	<---	Mother’s Education	0.046	0.216
Student Conduct	<---	School Year	−0.025	0.660
Study Behaviors	<---	School Year	0.018	0.736
Emotional Engagement	<---	School Year	−0.005	0.927
Cognitive Engagement	<---	School Year	−0.086	0.119
Teacher Support	<---	School Year	0.038	0.506
Family Support	<---	School Year	−0.155	0.005
Peer Support	<---	School Year	−0.035	0.541

Adapted from: M. A. S. M. Santos (2023). Retenção Escolar e Envolvimento dos Estudantes com a Escola: desafios para a educação inclusiva. Doctoral Dissertation. University of Vigo, Ourense Campus. Unpublished Dissertation.

**Table 2:** Unstandardized estimates (SE) of intercept and slope parameters (Fixed and Random Effects) in unconditional growth models for student engagement dimensions over time (longitudinal samples).

Dependent Variables	Fixed Effects (Mean)		Random Effects (Variances)	
	Intercept	Slope	Intercept	Slope
Student Engagement with school	3.27 (0.02) ***	−0.10 (0.02) ***	0.07 (0.01) ***	0.06 (0.01) ***
Student Conduct	3.37 (0.03) ***	−0.20 (0.03) ***	0.05 (0.02) **	0.05 (0.03) ^n.s.^
Study Behaviors	3.28 (0.05) ***	0.03 (0.06) ^n.s.^	0.37 (0.06) ***	0.37 (0.09) ***
Emotional Engagement	3.30 (0.03) ***	−0.35 (0.04) ***	0.09 (0.02) ***	0.10 (0.03) **
Cognitive Engagement	3.28 (0.03) ***	−0.21 (0.04) **	0.13 (0.02) ***	0.13 (0.03) ***
Teacher Support	3.01 (0.03) ***	0.01 (0.04) ^n.s.^	0.08 (0.02) ***	0.08 (0.04) ^n.s.^
Family Support	3.55 (0.03) ***	0.02 (0.04) ^n.s.^	0.16 (0.02) ***	0.14 (0.04) ***
Peer Support	3.12 (0.03) ***	−0.08 (0.04) *	0.11 (0.02) ***	0.12 (0.04) ***

Note. * *p* ≤ 0.05; ** *p* ≤ 0.01; *** *p* ≤ 0.001; n.s. = not significant. Adapted from: M. A. S. M. Santos (2023). Retenção Escolar e Envolvimento dos Estudantes com a Escola: desafios para a educação inclusiva. Doctoral Dissertation. University of Vigo, Ourense Campus. Unpublished Dissertation.

**Table 3:** Effect of retention on student engagement over time—standardized estimates of intercept and slope parameters (fixed and random effects) from the conditional growth models of the overall scale of student engagement with school and its seven dimensions.

Dependent Variables		Control Variables					Predictor Variables		
		Gender (Female)	Age_2_	Mother’s Education	School Year_2_	Retention_3_	Intercept–Slope Corr.	Mean	Variance
Student Engagement with school	Intercept	0.02 ns	—	0.15 ns	—	—	−0.36 ns	3.20 ***	0.49 ***
	Slope	0.12 ns	−0.05 ns	−0.03 ns	−0.07 ns	−0.29 *		0.34 ns	0.50 ***
Student Conduct	Intercept	0.24 *	—	0.06 ns	—	—	−0.07 ns	3.28 ***	0.04 **
	Slope †	—	—	—	—	—		−0.2 ***	0.05 ns
Study Behaviors	Intercept	−0.06 ns	—	0.05 ns	—	—	−0.63 **	3.30 ***	0.27 ***
	Slope	0.25 **	−0.03 *	0.08 ns	−0.23 *	−0.44 ***		1.42 ns	0.29 ***
Emotional Engagement	Intercept	−0.01 ns	—	−0.05 ns	—	—	−0.30 ns	3.34 ***	0.06 ***
	Slope	−0.03 ns	0.03 ns	−0.06 ns	0.23 ns	−0.09 ns		−0.79 ns	0.09 **
Cognitive Engagement	Intercept	0.00 ns	—	0.25 **	—	—	−0.51 **	3.14 ***	0.09 ***
	Slope	0.23 *	0.00 ns	−0.15 ns	0.01 ns	−0.09 ns		−0.19 ns	0.12 ***
Teacher Support	Intercept	−0.11 ns	—	0.09 ns	—	—	−0.12 ns	2.99 ***	0.07 **
	Slope †	—	—	—	—	—		0.01 ns	0.08 ns
Family Support	Intercept	0.09 ns	—	0.25 **	—	—	−0.34 ns	3.34 ***	0.09 ***
	Slope	−0.10 ns	0.04 ns	−0.06 ns	0.09 ns	−0.19 ns		−0.11 ns	0.11 **
Peer Support	Intercept	0.13 ns	—	0.06 ns	—	—	−0.41 *	3.04 ***	0.10 ***
	Slope	−0.25 *	−0.1 ns	0.04 ns	0.28 *	−0.25 ns		0.32 ns	0.11 **

Note. The values correspond to the standardized estimates and their statistical significance. † These parameters were not estimated because the variable did not exhibit significant variability around the slope. “—” These parameters were not estimated because the measurement lies independent variables lie after the first measurement point; Int: Intercept * *p* ≤ 0.05; ** *p* ≤ 0.01; *** *p* ≤ 0.001; ns = not significant (*p* > 0.05). Adapted from: M. A. S. M. Santos (2023). Retenção Escolar e Envolvimento dos Estudantes com a Escola: desafios para a educação inclusiva. Doctoral Dissertation. University of Vigo, Ourense Campus. Unpublished Dissertation.

## Data Availability

No new data were created or analyzed in this study. Data sharing is not applicable to this article.

## Appendix A

**Table A1:** Descriptive statistics of variables across the three measurement points of the longitudinal data.

Variable	Range	*M*	*SD*	Skew	Kurt
M1 Student Engagement with school	1–5	3.34	0.50	0.09	0.51
M2 Student Engagement with school	1–5	3.23	0.55	−0.17	0.04
M3 Student Engagement with school	1–5	3.32	0.55	−0.23	0.09
M1 Student Conduct	1–4	3.37	0.41	−0.37	2.20
M2 Student Conduct	1–4	3.19	0.49	−0.35	0.39
M3 Student Conduct	1–4	3.25	0.48	−0.50	0.96
M1 Study Behaviors	1–5	3.32	0.77	−0.01	0.04
M2 Study Behaviors	1–5	3.27	0.78	−0.23	0.00
M3 Study Behaviors	1–5	3.38	0.78	−0.32	0.19
M1 Emotional Engagement	1–4	3.27	0.40	−0.56	3.87
M2 Emotional Engagement	1–4	3.03	0.47	−0.04	0.55
M3 Emotional Engagement	1–4	3.01	0.45	−0.18	1.62
M1 Cognitive Engagement	1–4	3.18	0.43	−0.24	0.20
M2 Cognitive Engagement	1–4	3.12	0.47	−0.56	0.69
M3 Cognitive Engagement	1–4	3.10	0.43	−0.30	−0.04
M1 Teacher Support	1–4	2.95	0.50	−0.12	0.77
M2 Teacher Support	1–4	3.03	0.52	−0.48	1.27
M3 Teacher Support	1–4	3.06	0.48	−0.34	1.50
M1 Family Support	1–4	3.39	0.51	−0.63	0.50
M2 Family Support	1–4	3.60	0.47	−1.07	0.80
M3 Family Support	1–4	3.57	0.49	−0.99	0.84
M1 Peer Support	1–4	3.16	0.46	−0.48	1.55
M2 Peer Support	1–4	3.11	0.49	−0.32	0.95
M3 Peer Support	1–4	3.09	0.46	−0.23	0.70

## Appendix B

**Table A2:** Items used for measuring the seven dimensions of student engagement with school.

Multifactor Measure of Student Engagement—MMSE (Moreira et al., 2020a)				
Mark *1* if you *Totally Disagree* with the statement; Mark *2* if you *Disagree* with the statement; Mark *3* if you *Agree* with the statement; Mark *4* if you *Totally Agree* with the statement.	Totally Disagree	Disagree	Agree	Totally Agree
1. At my school, teachers care about students	1	2	3	4
2. My teachers are there for me when I need them	1	2	3	4
3. Overall, my teachers are open and honest with me	1	2	3	4
4. Most of my teachers care about how I’m doing	1	2	3	4
5. I check my schoolwork for mistakes	1	2	3	4
6. I study at home even when I don’t have a test	1	2	3	4
7. I talk with people outsider of school about what I am learning in class	1	2	3	4
8. I enjoy the work I do in class	1	2	3	4
9. What I’m learning in my classes will be important in my future	1	2	3	4
10. The grades in my classes do a good job of measuring what I’m able to do	1	2	3	4
11. The things I am learning in school are going to be importante to me in later life	1	2	3	4
12. School is important for achieving my future goals	1	2	3	4
13. I treat my teachers with respect	1	2	3	4
14. I respect most of my teachers	1	2	3	4
15. I treat my classmates with respect	1	2	3	4
16. I follow rules in school	1	2	3	4
17. I feel like I belong in my school	1	2	3	4
18. I feel close to people at my school	1	2	3	4
19. I am happy to be at my school	1	2	3	4
20. Students at my school are there for me when I need them	1	2	3	4
21. Other students at school care about me	1	2	3	4
22. Students here respect what I have to say	1	2	3	4
23. Other students here like me the way I am	1	2	3	4
24. when I have problems at school my family/guardian(s) are willing to help me	1	2	3	4
25. My family/guardian(s) are there for me when I need them	1	2	3	4
26. When something good happens at school, my family/guardian(s) want to know about it	1	2	3	4
27. My family/guardian(s) want me to keep trying when things are tough at school	1	2	3	4
